# A novel frameshift pathogenic variant in *ST3GAL5* causing salt and pepper developmental regression syndrome (SPDRS): A case report

**DOI:** 10.1038/s41439-021-00164-8

**Published:** 2021-08-12

**Authors:** Jamal Manoochehri, Seyed Alireza Dastgheib, Hossein Jafari Khamirani, Maryam Mollaie, Zahra Sharifi, Sina Zoghi, Seyed Mohammad Bagher Tabei, Sanaz Mohammadi, Fatemeh Dehghanian, Zahra Farbod, Mehdi Dianatpour

**Affiliations:** 1 Department of Genetics, Marvdasht Branch, Islamic Azad University, Marvdasht, Iran; 2 Department of Medical Genetics, Shiraz University of Medical Sciences, Shiraz, Iran; 3 Comprehensive Medical Genetic Center, Shiraz University of Medical Sciences, Shiraz, Iran; 4 Student Research Committee, Shiraz University of Medical Sciences, Shiraz, Iran; 5 Maternal-Fetal Medicine Research Center, Shiraz University of Medical Sciences, Shiraz, Iran; 6 Stem Cells Technology Research Center, Shiraz University of Medical Sciences, Shiraz, Iran

**Keywords:** Genetics research, Epilepsy

## Abstract

GM3 synthase deficiency is associated with salt and pepper developmental regression syndrome (SPDRS), a rare genetic disorder. Herein, we report the first Iranian patient with SPDRS. We detected a novel pathogenic variant of *ST3GAL5* (NM_003896.4: c.1030_1031del, p.Ile344Cysfs*11). The proband had intellectual disability (ID), failure to thrive, cerebral atrophy, microcephaly, and atonic seizures. The main future challenge proceeding from the results of this study is the prenatal detection of the newly discovered variant; the next step would involve further studies to elucidate the phenotypic spectrum of SPDRS and detect new variants that could cause symptoms ranging from mild to severe.

GM3 synthase is the first enzyme involved in the biosynthesis of a- and b-series gangliosides. Pathogenic variants in *ST3GAL5* that result in a complete lack of GM3 activity lead to the elimination of all of its downstream biosynthesis products. SPDRS, a rare neurological disorder caused by GM3 synthase deficiency, leads to a severe, early-onset neurological syndrome characterized by drug-resistant epilepsy, failure to thrive, and general motor and cognitive impairment^1,2^.

Here, we report the clinical features of the first Iranian patient with SPDRS; in this case, the disease was caused by a novel pathogenic variant (NM_003896.4: c.1030_1031del, p.Ile344Cysfs*11) of *ST3GAL5*. Written informed consent was obtained from each subject individually or, from the parents of the underage patient. This report is written in compliance with the CAse REport (CARE) Statement^3^.

The proband was a three-and-a-half-year-old girl who was referred to our center with global developmental delay. She was the only child born to consanguineous parents (Fig. 1A). An anomaly scan in the 18th week showed microcephaly. The delivery was uncomplicated. The proband had normal birth parameters; however, she showed subsequent regression. Routine growth check-ups revealed that she had failed to reach developmental milestones. To rectify the growth delay, increasing formula concentrations were administered. The caregivers were counseled on healthy, nutrient-rich food choices, and the timing of meals and snacks. Following the insufficient response, percutaneous endoscopic gastrostomy was carried out at the age of 2 years; this intervention was discontinued after the occurrence of a set of complications and poor adherence by the family. Afterward, the dietary interventions were resumed.

**Fig. 1:**
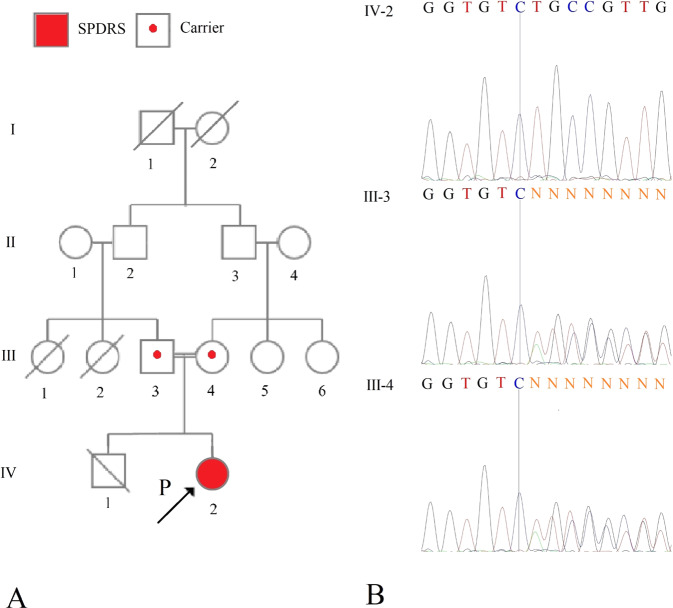
Pedigree and electropherogram of the proband and her parents. **A** Pedigree. **B** Electropherogram of the proband, her father, and her mother.

The first symptoms were recorded during the first 2 months of life, primarily comprising irritability, poor feeding, and failure to thrive. The family had previously had a son who died at the age of 4 years. The parents reported features suggesting SPDRS. However, the boy had not been rigorously followed up during his life, and no reliable data on his condition are currently available.

On physical examination, the proband was hypotonic and did not have full control of her head. Other motor skills had also failed to develop. The proband could not sit, roll over, or stand independently. She lay “frog-legged”, with adducted hips and flexed knees (characteristic of hypotonia). As a result, she was bedridden and entirely dependent on her parents for ambulation. The patient had not gained any weight in 6 months and was severely underweight for her age, at 8 kg and 90.5 cm tall (both under the fifth percentile). She also had microcephaly (head circumference 42.5 cm). The patient’s low weight, poor weight gain, and microcephaly suggested failure to thrive (classified as severe by the Gomez criteria). She had severe ID, in line with previous reports of SPDRS. The ophthalmological and auditory examination using brainstem auditory evoked potentials, slit-lamp examination, fluorescein angiography, noncontact tonometry, and optical coherence tomography did not detect any sensory impairment. However, the patient had visual and auditory tracking disorders that were noticed in qualitative chairside testing. These examinations suggest that the tracking disorder arose from disturbances in the central nervous system and a lack of coordination between various centers in the brain. Moreover, no language development was apparent, and the patient remained mute with poor nonverbal expression. She had also experienced mild atonic seizures a year earlier. Considering her unremarkable electroencephalography results a month after the seizure episodes, no medication was initiated for seizure control, and she was merely followed up. She did not have any gastrointestinal disorders, but she needed feeding assistance. On brain imaging, the prominence of extra-axial spaces filled with cerebrospinal fluid around the cerebral hemispheres indicated mild brain atrophy. Otherwise, the anatomy of the brain was normal (Fig. 2).

**Fig. 2:**
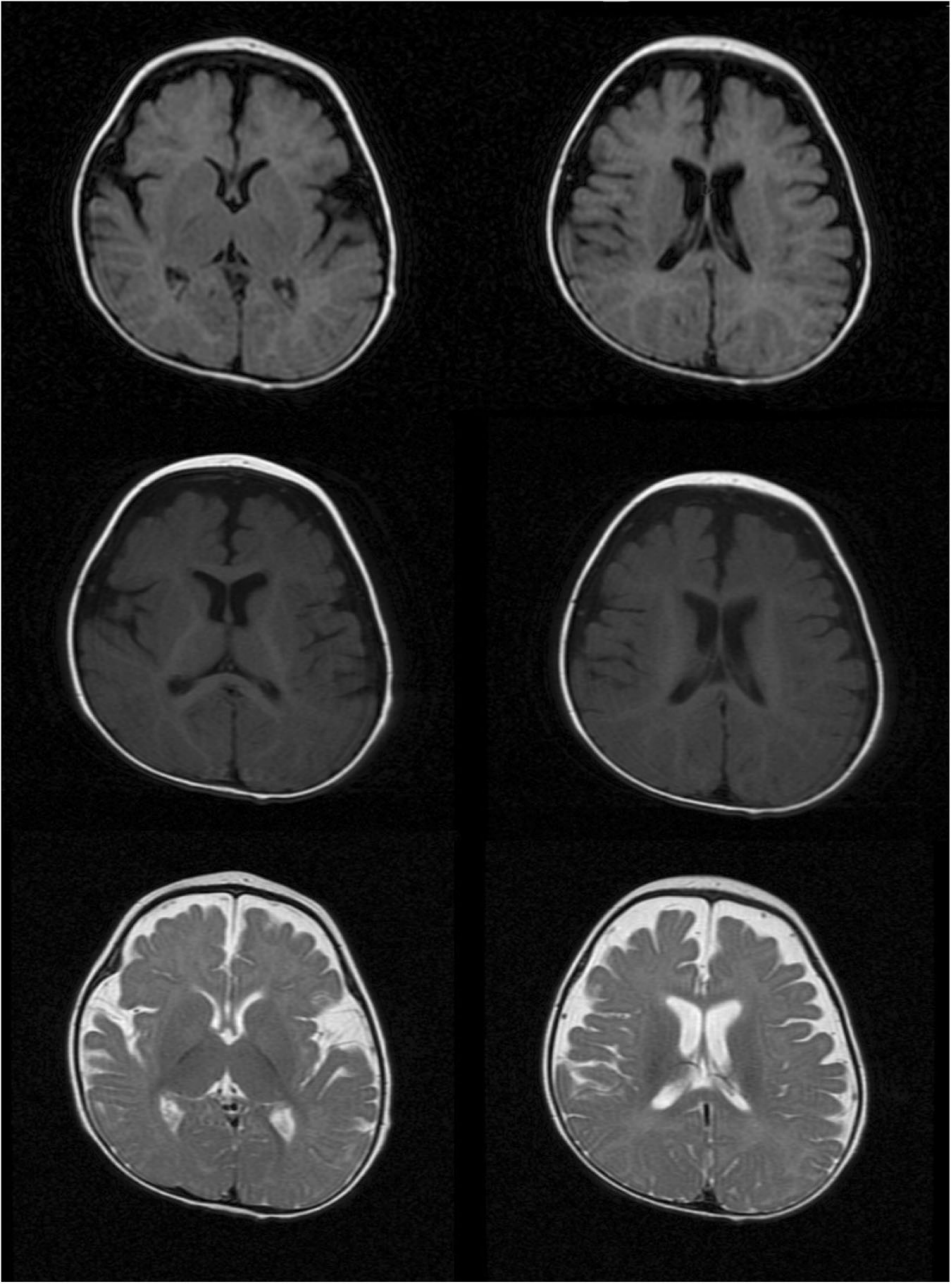
Brain MRI of the proband. Magnetic resonance imaging suggests mild cerebral atrophy (conducted at 6 months of age).

The cardiovascular assessment was unremarkable. The patient had never had any skin disorder (no pigment change or ichthyosis). She also had no dysmorphic craniofacial features except for microcephaly. No gross anatomical anomaly was observed in the extremities.

Prior to whole-exome sequencing (WES), she underwent a set of laboratory tests for congenital metabolic disorders. No abnormality was noticed in neonatal screening results, amino acid metabolism, beta-oxidation of fatty acids, or carnitine metabolism. The urea cycle, acylcarnitine levels, and organic acid profile were normal. However, galactose-1-phosphate uridylyltransferase was elevated on one occasion.

Using a QIAamp DNA Blood Mini Kit (Qiagen, Hilden, Germany), genomic DNA was extracted from peripheral white blood cells for WES, Sanger sequencing, and further investigations. Genomic DNA was captured using the SureSelect XT Human All Exon V6 reagent kit (cat. no. 5190-8863; Agilent Technologies, Inc.), which was applied for the enrichment of coding exons and flanking intronic sequences in accordance with the manufacturer’s instructions. Captured coding DNA samples were sequenced using an Illumina NovaSeq6000 (Illumina San Diego, CA) with 100-bp paired-end sequencing. The raw data were aligned against the human reference genome (hg19) using the Burrows-Wheeler Aligner^4^. Single-nucleotide polymorphisms (SNPs) were called by the software program Genome Analysis Toolkit (GATK). Variants were annotated using ANNOVAR^5^. Each variant was classified into one of five categories, namely, pathogenic, likely pathogenic, variant of unknown significance (VUS), likely benign, and benign, based on the ACMG standards for the interpretation of sequence variations^6^. The phenotypic features associated with the candidate genes were compared with the patient’s phenotype. Core phenotypes of the variants were obtained from the OMIM database and utilized to acquire a gene list for a virtual panel using the OMIM database (OMIM #609056).

WES identified a homozygous frameshift pathogenic variant (NM_003896.4: c.1030_1031del, p.Ile344Cysfs*11) of *ST3GAL5* in the proband; this variant was also found in both parents in a heterozygous state. Pathogenic variants of *ST3GAL5* are associated with SPDRS. The variant detected by WES is a frameshift variant and is classified as “pathogenic” based on the PVS1, PM2, PM3, and PP3 criteria of the ACMG/AMP guidelines. Null variants of *ST3GAL5*, including loss-of-function variants, are a known disease mechanism associated with SPDRS. The variant was not found in gnomAD genomes, exomes, or ClinVar.

We confirmed the presence of this variant by Sanger sequencing (Fig. 1B). The primers were designed using Oligo Primer Designer6 (Supplemental Table S1).

Glycosphingolipids (GSLs) represent a large group of molecules that play essential roles throughout the body, especially the brain. Qualitative and quantitative changes in ganglioside expression in the nervous system correlate with certain cellular events during development. Thus, they are inferred to play regulatory roles in the developing nervous system^7^. In the formation of glycosphingolipids, the enzyme ST3 beta-galactoside alpha-2,3-sialyltransferase 5 (ST3GAL5) converts lactosylceramide into GM3 ganglioside, the biosynthetic precursor to other complex ganglioside molecules, many of which have significant roles in the development of neural tissue^8^. Defects in ST3GAL5 in humans have previously been linked to a spectrum of clinical presentations. GM3 synthase deficiency was initially described as “salt and pepper syndrome” in a family with pigmentary changes and ID and was later described in eight children from an Old Order Amish family with failure to thrive, epilepsy, profound developmental regression, and quadriplegic cerebral palsy^8–10^. Several other reports have noted additional clinical features including blindness, deafness, neurocutaneous disorders, and growth failure^8,10,11^.

This paper reports what is, to the best of our knowledge, the first Iranian case of SPDRS caused by a novel pathogenic variant in *ST3GAL5*. The clinical features described in this paper are consistent with the previously described phenotypes. However, a few features presented by this patient are uncommon, e.g., atonic seizures.

Iran is located in the consanguinity belt, stretching from North Africa to South India^12^. This high rate of closely consanguineous marriages brings about a set of genetic disorders that are extremely rare individually but abundant as a group. The ST3GAL family is a good case in point. We recently reported a patient with a pathogenic variant in *ST3GAL3*^13^. Pathogenic variants in both *ST3GAL5* and *ST3GAL3* are extremely rare; however, these reports show that the total number of cases of these two disorders is considerable.

Concerning the importance of the pathogenic variants of *ST3GAL5* and the phenotypic spectrum of patients affected by SPDRS, this newly discovered variant needs to be actively screened for, studied, and clinically characterized. The main future challenge proceeding from the results of this study is the prenatal detection of the newly discovered variant; the next step would involve further studies to elucidate the phenotypic spectrum of SPDRS and detect new variants that could cause symptoms ranging from mild to severe. With such information, decisions to abort or continue the pregnancy can be made on a more solid basis in the future.

## HGV Database

The relevant data from this Data Report are hosted at the Human Genome Variation Database at 10.6084/m9.figshare.hgv.3075.

## Supplementary information

Supplementary Material Table 1

Supplementary Material Table 2

## Data Availability

All data generated or analyzed during this study are included in the final published article.
