# Correction: A novel frameshift pathogenic variant in *ST3GAL5* causing salt and pepper developmental regression syndrome (SPDRS): a case report

**DOI:** 10.1038/s41439-021-00174-6

**Published:** 2021-11-22

**Authors:** Jamal Manoochehri, Seyed Alireza Dastgheib, Hossein Jafari Khamirani, Maryam Mollaie, Zahra Sharifi, Sina Zoghi, Seyed Mohammad Bagher Tabei, Sanaz Mohammadi, Fatemeh Dehghanian, Zahra Farbod, Mehdi Dianatpour

**Affiliations:** 1 Department of Genetics, Marvdasht Branch, Islamic Azad University, Marvdasht, Iran; 2 Comprehensive Medical Genetic Center, Shiraz University of Medical Sciences, Shiraz, Iran; 3 Department of Medical Genetics, Shiraz University of Medical Sciences, Shiraz, Iran; 4 Student Research Committee, Shiraz University of Medical Sciences, Shiraz, Iran; 5 Maternal-Fetal Medicine Research Center, Shiraz University of Medical Sciences, Shiraz, Iran; 6 Stem Cells Technology Research Center, Shiraz University of Medical Sciences, Shiraz, Iran

**Keywords:** Genetics research, Epilepsy

Correction to: *Hum Genome Var* 10.1038/s41439-021-00164-8, published online 12 August 2021

In this article the affiliation details for Jamal Manoocheri were incorrectly given as ‘Comprehensive Medical Genetic Center, Shiraz University of Medical Sciences, Shiraz, Iran’ but should have been ‘Department of Genetics, Marvdasht Branch, Islamic Azad University, Marvdasht, Iran’.

The original article has been corrected.

