# Supplementary material for: A novel frameshift pathogenic variant in ST3GAL5 causing salt and pepper developmental regression syndrome (SPDRS): A case report
Source: Hum Genome Var. 2021 Aug 12;8:33. doi: 10.1038/s41439-021-00164-8 (PMC8361121; doi:10.1038/s41439-021-00164-8)
Supplement: Supplementary file 1 — Supplementary Material Table 1 [file 41439_2021_164_MOESM1_ESM.docx]

| ST3GAL5 primers | Forward primer | Reverse primer |
| --- | --- | --- |
| Sequence | TGAGCTGCACTTCAAAGTA | AGACCATGTTTTAGAGCCTA |

Supplementary Material Table 1. The sequence of the forward and reverse primers utilized for Sanger sequencing.
