# Supplementary material for: A novel frameshift pathogenic variant in ST3GAL5 causing salt and pepper developmental regression syndrome (SPDRS): A case report
Source: Hum Genome Var. 2021 Aug 12;8:33. doi: 10.1038/s41439-021-00164-8 (PMC8361121; doi:10.1038/s41439-021-00164-8)
Supplement: Supplementary file 2 — Supplementary Material Table 2 [file 41439_2021_164_MOESM2_ESM.docx]

| Number of Overlapping Phenotypic Features | Genes |
| --- | --- |
| 4 | ABCD1, SCN1A, ABCC8, PTS, SURF1, BTD, NPC1, GCH1, ASL, CDKL5, ASS1, ATM, PRF1, GAMT, PDHA1, CPS1, OFD1, PLA2G6, SOX10, ETHE1, GJA1, ADSL, PROKR2, FGFR1, PPT1, FKRP, OTX2, POMGNT1, NPC2, SC02, SIL1, BBS2, UNC13D, POMT1, TBX1, BBS1, STXBP1, BBS10, NDUFS4, ALMS1, GJC2, STXBP2, NPHP1, BRAF, HESX1, NDUFV1, ECHS1, MKKS, ERCC8, GMPPB, BBS12, NDUFS8, TUBB2B, POLR1C, COQ2, MKS1, SUCLG1, FMR1, BBS4, POLR3B, SPR, RAB3GAP1, GBA, PUG, GP1BB, HSD17B4, PEX1, ERCC2, BCS1L, DOCK8, PEX10, TCF4, PEX12, ERCC6, RRM2B, PEX26, PEX2, ERCC4, PEX16, GRIN2B, PEX5, ERCC1, WDR73, PEX3, K1F1A, PEX14, PEX19, PEX11B, ADGRG1, C100RF2, ADLH5A1, RAF1, NDUFAF2, SDHA, EDNRB, CC2D2A, RARS2, ARL6, TSEN54, SUOX, SLC17A5, MBD5, POMT2, SCN2A, PMM2, CEP290, ST3GAL5, MMADHC, SCN9A, MFSD8, NDUFS2, SLC25A1, BBS7, POLR3A, PCNT, NDUFS6, EDN3, PDHX, PNKP, BBS9, WWOX, PSAP, DPMI, DYRK1A, NDUFA1, PET100, TTC8, ALG6, FKTN, DLD, NDUFS1, TMEM216, BBS5, SDCCAG8, SLC19A3, SYNGAP1, HIBCH, NDUFS7, COX6B1, NDUFAF1, MTFMT, SLC6A19, ALG1, LARGE, ERCC3, NOTCH1, CTC1, KCNJ10, GLI2, IFT172, TRIM32, NDUFS3, LIPT1, DOCK6, DYNC1H1, NDUFAF3, SCO1, NDUFB9, SLC46A1, NDUFA2, TMEM138, NDUFB3, DLL4, NDUFAF5, TTC19, GABRA1, COA3, FOXRED1, STX11, COX10, SLC25A4, DEAF1, ACO2, NDUFV2, B3GALNT2, GRIN1, APOPT1, NUBPL, TSFM, CDH15, NDUFA12, CYC1, WDPCP, RAB3GAP2, RFT1, TACOI1, COX14, TMEM231, TMEM237, NDUFA11, GRM1, NDUFAF6, ZNF423, RPIA, KIRREL3, ATP5A1, NDUFA4, IFT27, COMT, PDSS2, NDUFAF4, UQCC2, LZTFL1, EOGT, UQCRQ, NDUFA9, COX15, NDUFA10, UQCRC2, UQCC3, DHFR, BBIP1, PDP1, CACNG2, PLXND1, COX20, ARHGAP31, RBPJ, EPB41L1, NIN, CTDP1, MY05A, UQCRB, NAT8L, LYRM7, FASTKD2, ZNF592, C50RF42, ND3, ND2, TRNV, ND5, ND4, ND1, ATP6, CYTB, ND6, TRNL1, COX2, COX3, TRNK, RNU4ATAC, COX1, TRNW |
| 3 | OTC, DMD, PROC, SDHB... |
| 2 | GLA, PAH, GCK, GALT... |
| 1 | HBB, LDLR, MLHI ... |

Supplementary Table 2
